# Pay-Day

**Published:** 1891-04

**Authors:** 


PAY-DAY.—We hope, our friends will bring along an extra $2 bill with them to Waco, to pay for the JOURNAL; and we ask all our old friends and subscribers to interest themselves in getting their professional friends to take the JOURNAL. If three or more will club their subscriptions, a discount of one-fourth, or 50 cents, will be allowed on each. It is impossible to put a sample copy into the hands of every physician; and it is only necessary for an intelligent non-subscriber to see the JOURNAL, to appreciate the fact, that a strenuous effort is being made in behalf of legitimate medicine, and the advancement of medical science in Texas, and he will subscribe at once. Nothing succeeds so well in getting subscriptions as personal solicitation. Show your JOURNAL to your friends, doctor, and ask them to join you in making up a club. The JOURNAL is worthy of support; it has been pronounced by the entire medical press "*creditable to the profession* in a high degree."

A gratifying indication is the fact that recently the JOURNAL, without solicitation, has been ordered by a number of the most prominent north Texas physicians. They wish to keep up with the music of progress.

